# Genome sequence analysis of the fairy ring-forming fungus *Lepista sordida* and gene candidates for interaction with plants

**DOI:** 10.1038/s41598-019-42231-9

**Published:** 2019-04-10

**Authors:** Tomoyuki Takano, Naoki Yamamoto, Tomohiro Suzuki, Hideo Dohra, Jae-Hoon Choi, Yurika Terashima, Koji Yokoyama, Hirokazu Kawagishi, Kentaro Yano

**Affiliations:** 1 Bioinformatics Laboratory, School of Agriculture, Meiji University, 1-1-1 Higashi-Mita, Kawasaki, 214-8571 Japan; 2 Center for Bioscience Research and Education, Utsunomiya University, 350 Mine-machi, Utsunomiya, Tochigi 321-8505 Japan; 3 Research Institute of Green Science and Technology, Shizuoka University, 836 Ohya, Suruga-ku, Shizuoka 422-8529 Japan; 4 Graduate School of Integrated Science and Technology, Shizuoka University, 836 Ohya, Suruga-ku, Shizuoka 422-8529 Japan; 5 Graduate School of Science and Technology, Shizuoka University, 836 Ohya, Suruga-ku, Shizuoka 422-8529 Japan; 6 Present Address: Rice Research Institute, Sichuan Agricultural University, 211 Huiminglu, Wenjiang, Chengdu China

## Abstract

Circular patterns called “fairy rings” in fields are a natural phenomenon that arises through the interaction between basidiomycete fungi and plants. Acceleration or inhibition of plant vegetative growth and the formation of mushroom fruiting bodies are both commonly observed when fairy rings form. The gene of an enzyme involved in the biosynthesis of these regulators was recently isolated in the fairy ring-forming fungus, *Lepista sordida*. To identify other genes involved in *L. sordida* fairy ring formation, we used previously generated sequence data to produce a more complete draft genome sequence for this species. Finally, we predicted the metabolic pathways of the plant growth regulators and 29 candidate enzyme-coding genes involved in fairy-ring formation based on gene annotations. Comparisons of protein coding genes among basidiomycete fungi revealed two nitric oxide synthase gene candidates that were uniquely encoded in genomes of fairy ring-forming fungi. These results provide a basis for the discovery of genes involved in fairy ring formation and for understanding the mechanisms involved in the interaction between fungi and plants. We also constructed a new web database F-RINGS (http://bioinf.mind.meiji.ac.jp/f-rings/) to provide the comprehensive genomic information for *L. sordida*.

## Introduction

**Figure 1:**
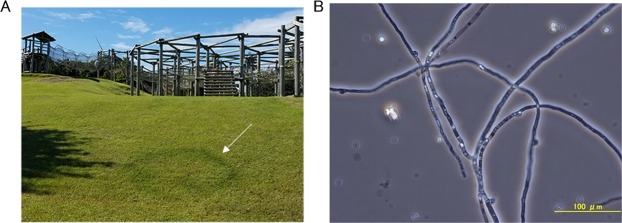
Photographs of fairy rings and *Lepista sordida*. (**A**) Fairy rings on turfgrass. White arrow indicates fairy ring with circular shape. (**B**) Micrograph of *L. sordida* mycelia. The scale bar indicates 100 μm.

Fairy rings are a natural phenomenon that exhibits circular patterns on turfgrass, pasture, or meadows due to interaction between basidiomycete fungi and plants^1^. When a fairy ring forms, plant growth acceleration or inhibition is observed, followed by formation of fungal fruiting bodies (Fig. 1A)^2^. More than 50 mushroom-forming fungi in several genera are known to form fairy rings^3,4^. In studies of interaction between fungi and the plants, Shantz and Piemeisel described that fungi degrade proteins in withered grass or soil into nitrogenous compounds, which can be easily utilized by plants, stimulating plant growth^5^. Recently, Xing *et al*. revealed the changes in soil components and microbial distribution during the fairy ring formation by *Floccularia luteovirens*^6^.

One of the fairy ring-forming fungi, *Lepista sordida*, belongs to the Tricholomataceae^7^. This fungus is distributed in lowland forests, lawns, and farmlands in the temperate zone of the northern hemisphere and forms pale purple mushrooms. We have reported the causative chemicals, 2-azahypoxanthine (AHX) and imidazole-4-carboxamide (ICA), for fairy ring formation by this fungus^8,9^. Furthermore, we isolated a derivative of AHX, 2-aza-8-oxohypoxanthine (AOH), in plants^10^. These compounds have been named “fairy chemicals”^11^ and can regulate growth of all tested plant species, including turfgrass, rice, potato and wheat^8,9,12^. We also reported that fairy chemicals were endogenously synthesized in plants by a novel purine metabolic pathway^10^. Based on the results above, we have hypothesized that fairy chemicals are a new family of plant hormones^13,14^. It appears that adenine/5-aminoimidazole-4-carboxamide phosphoribosyltransferase (APRT), which catalyzes the reaction from 5-aminoimidazole-4-carboxamide ribonucleotide (AICAR) to 5-aminoimidazole-4-carboxamide (AICA), provides a precursor of AHX^15^. Since AICAR is an intermediate in the *de novo* purine nucleotide pathway, regulation of this pathway is presumably involved in fairy ring formation. However, less knowledge has accumulated on which genes are responsible at each step in this biological pathway and which genes are related to biosynthesis of the bioactive compounds. Elucidation of the mechanisms underlying fairy ring formation might lead to a better understanding of how fungi and plant species interact in nature and how plant species regulate their vegetative growth.

Due to the accumulation of extensive sequencing data, utilization of omics information has become the gold standard approach in biology^16–19^. Genome sequencing data in *L. sordida* were generated for identification of *APRT* in our previous study^15^. However, the data remain poorly characterized. In the present study, to expand genetic information related to formation of fairy rings, we analyzed the draft genome sequence in *L. sordida*. The genome sequencing data of this species obtained from Illumina and Roche 454 sequencers^15^ were used to construct a refined genome assembly. Our data analyses revealed general genomic features and a set of candidate genes for enzymes that could be involved in biosynthesis of the plant growth regulators. This report provides a basis of research on fairy ring formation, the biosynthetic pathway for the plant growth regulators, and interaction between fungi and plant species. The genome information resource has been released on the F-RINGS web database (http://bioinf.mind.meiji.ac.jp/f-rings/).

## Results and Discussion

### Features of the *L. sordida* genome

A finer draft genome assembly was constructed by hybrid assembly with better-optimized conditions than those of Suzuki *et al*.^15^. This time, we used the Illumina reads together with the longer reads of the GS FLX Titanium system with two options in the Newbler assembler to take into consideration of the genome size of this fungus and the heterogeneity of its genome. A total of 703 genomic scaffolds were generated (Table 1), fewer than the previous 812^15^. The ratio of ambiguous sequences in the genome assembly was lowered from 5.3% to 1.9%. Moreover, the N50 length was 398.6 kbp, considerably longer than the previously reported value of 178.9 kb. The draft genome sequence was comprised of 39.0 Mbp nucleotides with 1,476 gaps. The reliability of this genome assembly was tested using BUSCO, confirming that 95.7% of common gene sequences in eukaryotes were covered completely. The GC content of 44.9% was close to that in *Lentinula edodes*^20^, which is also a mushroom-forming fungus categorized into the Tricholomataceae, as with *L. sordida*. Sequences of the genomic scaffolds of *L. sordida* have been deposited at DNA Data Bank of Japan (DDBJ) (GenBank accession: BIMQ01000001-BIMQ01000703).

**Table 1:** Summary of genome assembly and annotation.

Assembly and annotation statistics	
Number of genomic scaffolds	703
N50 length (bp)	398,572
Total number of nucleotides of genomic scaffolds (bp)	39,026,461
Length of the longest scaffold (bp)	1,651,988
Length of the shortest scaffold (bp)	2,004
Average length of the scaffods (bp)	55,514
Ratio of ‘N’ (%)	1.9
GC content (%)	44.9
tRNA genes	194
rRNA genes	3
Number of protein coding genes*	11,823
Average gene length (bp)	1,580.7
Average exon length (bp)	222.8
Average number of exons per gene	7.0
Number of genes annotated by sequences in UniProt	6,468
Number of genes annotated by sequences in nr database	10,821
Number of genes annotated by KEGG pathway	1,878
Number of genes annotated by Gene Ontology	5,841
Number of genes with no annotation	91

*At least 100 amino acids long.

A *k*-mer analysis was performed to predict the genome size and assess genome heterogeneity. This analysis used the paired-end (PE) reads and mate paired-end (MP) reads separately. The PE reads provided the predicted genome size of 52 and 50 Mbp by JELLYFISH and GCE, respectively, suggesting that approx. 75% of the *L. sordida* genome is covered by the genomic scaffolds (Table 1). The analysis identified the major peak and a shoulder peak of *k*-mer frequencies, suggesting heterogeneity of the genome (Supplementary Fig. 1A). Analysis of the MP reads provided similar observations also suggesting heterogeneity of the genome (Supplementary Fig. 1B).

### Genes in the *L. sordida* genome

Genes on the genomic scaffolds were predicted by bioinformatic characterization. In this process, 3.0% of the genomic sequences were excluded from the subsequent gene searches because of repeat regions, the most abundant sequences of which were simple repeats and long terminal repeat elements. As summarized in Table 1, 194 tRNA genes, three rRNA genes and 11,823 protein coding genes were found. The 183 tRNA genes corresponded to the full set of 20 amino acids, while 11 were defined as pseudo tRNA genes by a tRNAscan-SE search. For the protein coding genes, the number of genes was typical as a basidiomycete fungus^21^. The following searches of the protein coding genes assigned 99.3% of the genes in the *L. sordida* genome to existing functional annotations. BLAST searches of the protein coding genes showed significant similarities of 91.5% (10,821) to sequences in the non-redundant protein database ‘nr’ and 54.7% (6,468) to sequences in the UniProtKB database.

**Figure 2:**
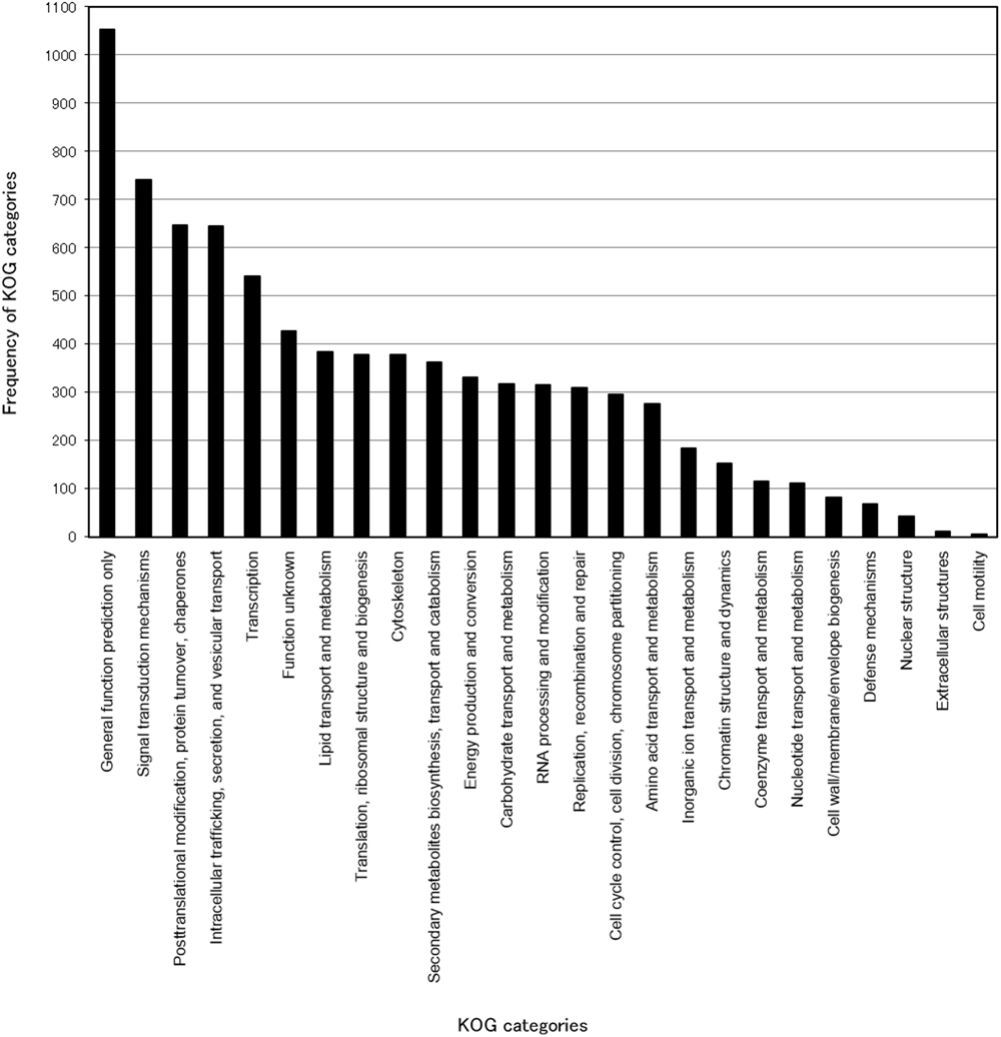
KOG categories in *L. sordida*. KOG annotations are given with the default parameter settings of the WebMGA web server. The vertical axis represents the frequency of each KOG class annotated out of all *L. sordida* proteins.

Some typical genes in mushroom-forming fungi such as those encoding lectins and hydrophobins, and genes for enzymes involved in lignocellulose degradation such as manganese peroxidases, cellobiose dehydrogenases, and laccases were found (Supplementary Table 1). An extensive survey revealed presence of various types of enzyme families for lignocellulose degradation: auxiliary activity families, glycoside hydrolase families, glycosyltransferase families, polysaccharide lyase families, carbohydrate esterase families, and carbohydrate-binding module families (Supplementary Table 2). These results are consistent with *L. sordida* being a white rot mushroom fungus. A KAAS search assigned KEGG orthology terms to 1,878 genes and an InterProScan search resulted in protein domain annotations for 5,841 genes. A total of 83 genes were assigned to the ‘Purine metabolism’ [map00230] category of the KEGG metabolic pathways, which could be relevant to fairy ring formation in *L. sordida*. Moreover, 23 genes were assigned to GO terms related to purine metabolism; for example, ‘purine ribonucleoside monophosphate biosynthetic process’ [GO:0009168] and ‘purine nucleotide biosynthetic process’ [GO:0006164]. KOG classification revealed the gene repertoire in the fungus (Fig. 2). The most abundant KOG class was ‘Signal transduction mechanisms’ (740 proteins, 5.9%), followed by ‘Posttranslational modification of proteins, protein turnover, chaperones’ (647 proteins, 5.1%) and ‘Intracellular trafficking, secretion, and vesicular transport’ (644 proteins, 5.1%). Total of 263 genes were annotated as transcription factors; for example, 15 genes for ‘C_2_H_2_-type Zn-finger protein’ [KOG2462], 14 genes for ‘HMG-box transcription factor’ [KOG0527], and 6 genes for ‘bHLH Zip transcription factor BIGMAX’ [KOG1319]. Pelkmans *et al*. reported that a C2H2-type Zn-finger protein gene *c2h2* in *Schizophyllum commune* is involved in mushroom formation^22^. Ait Benkhali *et al*. documented the role of HMG-box transcription factors in sexual reproduction in a fungus *Podospora anserine*^23^. Several key secretary protease genes for colonization, *aspartate proteases*, *extracellular metalloproteases*, *fungalysins*, and *subtilisins*, were also predicted (Supplementary Table 3). These functional annotations are accessible at ‘F-RINGS’ (see the section ‘Data access from F-RINGS web database’).

### Candidate genes for plant growth regulators involved in fairy ring formation

**Figure 3:**
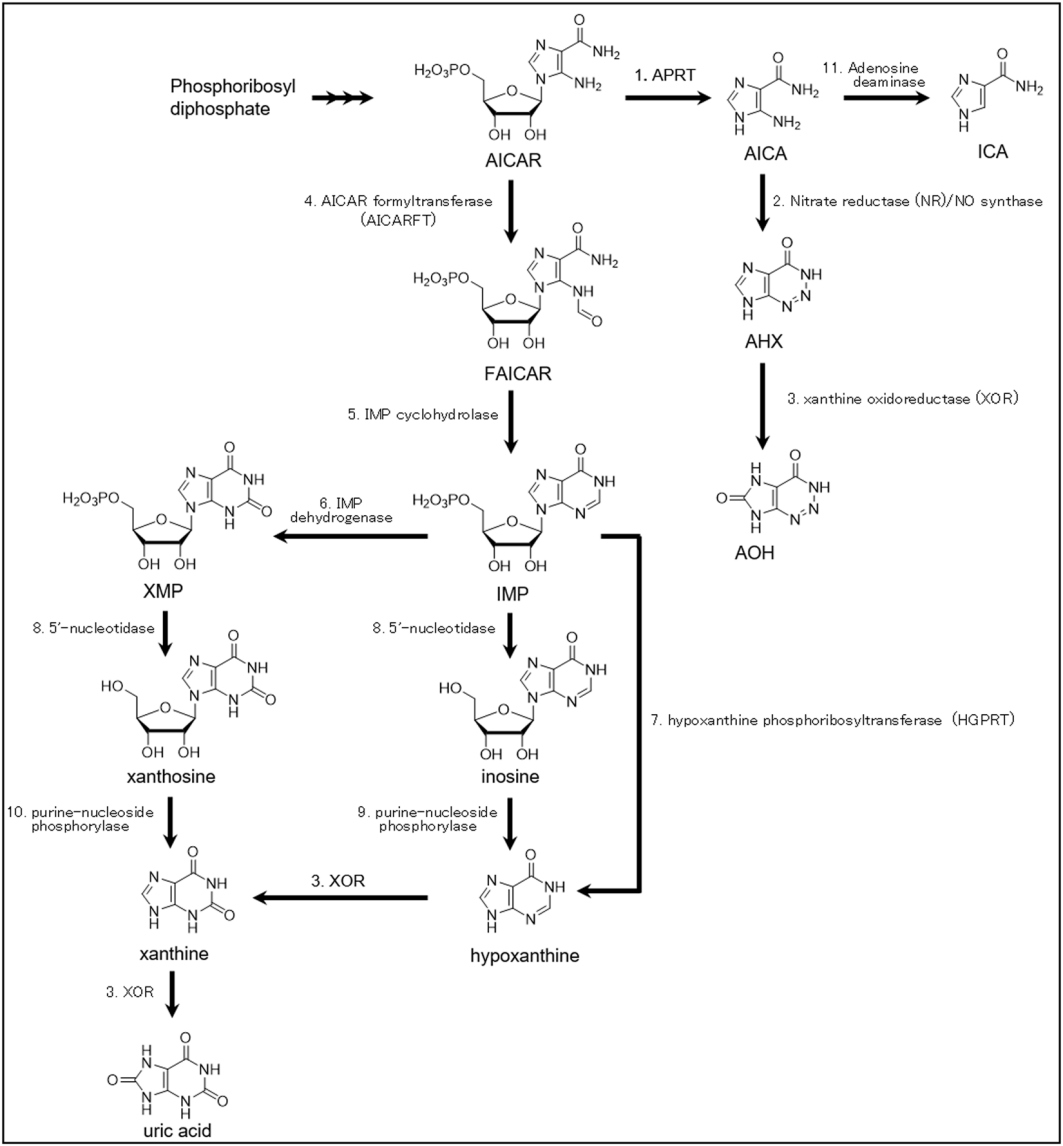
The biosynthetic pathway of AHX, AOH and ICA. 1: APRT, 2: NR or NO synthase, 3: XOR, 4: AICARFT, 5: IMP cyclohydrolase, 6: IMP dehydrogenase, 7: HGPRT, 8: 5′-nucleotidase, 9: purine nucleoside phosphorylase, 10: purine nucleoside phosphorylase. 11: adenosine deaminase.

Based on the biochemical and chemical basis of AHX and its derivatives, we hypothesized that their biosynthetic pathways are related to fairy ring formation and assigned probable enzymes for each reaction step (Fig. 3). AICAR, which is a metabolite in purine metabolism, is the precursor for synthesis of AHX and the derivative AOH in the predicted metabolic pathway. AICAR is probably converted into AICA by APRT^15^. The step from AICA to AHX was biochemically elusive^10^; however, it might incorporate the nitrogen of nitrite or nitric oxide into AICA in a diazo reaction followed by an intramolecular cyclization reaction between the nitrogen atoms. For this reason, we assumed that two enzymes, nitrate reductase (NR) and nitric oxide (NO) synthase, are involved in provision of the nitrogen atom for this biochemical step. Catalysis of AHX to AOH by xanthine oxidase (XOD) was proved by the biochemical assay of Choi *et al*.^10^. Since XOD is also a xanthine oxidoreductase (XOR), we assigned XOR to this reaction step. In chemical synthesis, AICA can be converted into ICA by diazotization followed by a reduction reaction^8^. Since a small amount of ICA was detected in cell extracts, AICA might be converted into ICA biochemically by adenosine deaminase or its homologues. Aside from those, AICAR is metabolized in the *de novo* purine nucleotide pathway by AICAR formyltransferase (AICARFT) and inosine monophosphate (IMP) cyclohydrolase (FAICAR) into IMP. IMP can be converted into xanthine via three biochemical reactions: via xanthosine, inosine and/or hypoxanthine. Finally, uric acid is synthesized. The pathway from AICAR to uric acid and to AOH can be competitive in regulation of metabolic flux.

We identified 29 enzyme-coding genes in the hypothesized pathway (Fig. 3; Supplementary Table 4). For biosynthesis of AHX and AOH, one NR and seven NO synthase genes were found as candidates. No XOD genes were found in *L. sordida*. Instead, we mined six genes for xanthine dioxygenase (XDO) based on the gene annotations. XDO can catalyze both hydroxylation of hypoxanthine to xanthine and hydroxylation of xanthine to uric acid^24,25^. XDO is a fungal enzyme that lacks a molybdopterin cofactor but is dependent on α-ketoglutarate and has the TauD motif^25^. For conversion from AICA to ICA, five genes annotated as adenosine deaminases might be candidates in the *L. sordida* genome; however, these genes need to be biochemically evaluated. In the branched pathway for biosynthesis of uric acid, genes encoding two AICARFTs, two IMP cyclohydrolases, one IMP dehydrogenase, one hypoxanthine phosphoribosyltransferase (HGPRT), one 5′-nucleotidase, and one purine nucleoside phosphorylase, a homologue of which is subject to allosteric regulation^26^, were mined.

### Genes common to species involved in fairy ring formation

**Figure 4:**
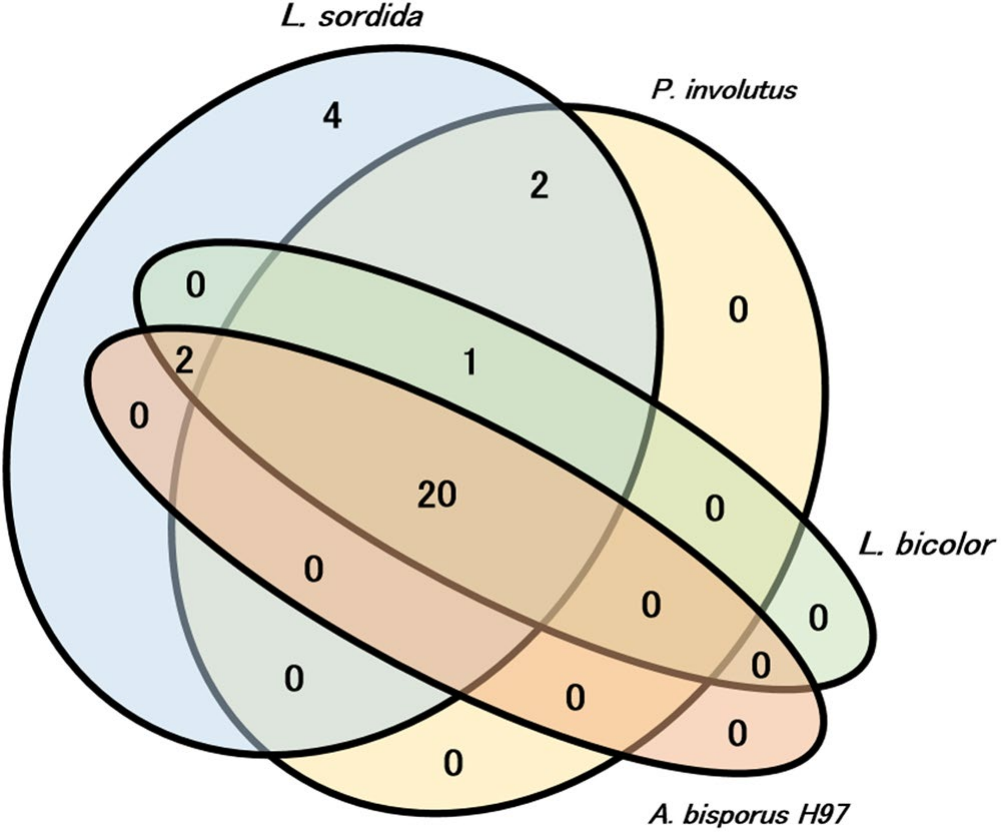
Conservation of gene candidates for biosynthesis of plant growth regulators among *P. involutus*. *L. bicolor*, *A. bisporus* and *L. sordida*. Numerals in the venn diagram show the number of genes in each category.

Comparison of genomic information from multiple species allows screening of genes that are uniquely conserved among species with the same biological function^27–30^. The genome of another fairy ring-forming fungus, *Paxillus involutus*, was sequenced by Kohler *et al*.^31^. In our phylogenetic analysis using the small subunit of the 18S ribosomal RNA gene, *L. sordida* and *P. involutus* seemed relatively divergent among the 48 basidiomycete fungi examined (Supplementary Fig. 2). In addition, *P. involutus* is a mycorrhiza, so would presumably have a characteristic set of genes distinct from the rot fungus *L. sordida*. If the mechanism of fairy ring formation is common in the two species, *P. involutus* should have homologues of the gene candidates in *L. sordida*. Hence, we looked for homologues in *P. involutus*. It appeared that 19 of the candidate genes, which cover all 10 reactions described above, have homologues in *P. involutus* (Fig. 4; Supplementary Table 4). Most of the 19 genes also had homologues in *Laccaria bicolor* and *Agaricus bisporus*, which do not form fairy rings as far as we know (Supplementary Table 4). Notably, in the two fairy ring-forming fungi, multiple NO synthase gene homologues were found, while only one NO synthase gene homologue was found in *L. bicolor* and *A. bisporus*. This result arose an idea that the additional NO synthase genes were the key factors. Gene expressions in a set of RNA-Seq data of *P. involutus* supported that 15 candidate genes were not likely to be pseudogenes, while the two additional NO synthases were expressed at lower levels than that of the conserved NO synthase genes (Supplementary Fig. 3). It remains to be examined whether such additional NO synthase genes are biologically significant or not.

We further attempted to screen for genes common in *L. sordida* and *P. involutus*, but not found in other mushroom-forming fungi, as candidates involved in fairy ring formation. BLASTP searches of all the protein sequences in *L. sordida* and *P. involutus* against 467 mushroom-forming fungi with an e-value of 1e^−5^ were conducted. Only two proteins were common in the two species uniquely: ‘snap-scaffold25|size362950-processed-gene-0.249-mRNA-1’ and ‘genemark-scaffold473|size3073-processed-gene-0.1-mRNA-1’. The functions of these proteins are unknown, though the latter protein had a transmembrane domain, suggesting that it is an integral membrane protein. This observation has three potential implications: (1) either or both of these genes are the key components regulating fairy ring formation, (2) at least one of these other 467 species can form fairy rings, and the inclusion of this species interfered with discovery of gene candidates for fairy ring formation (3) causal genes for fairy ring formation are partially or completely conserved across fairy ring-forming fungi and non-fairy ring-forming fungi, but gene expression or biochemical function are different between those two groups. However, it is possible that key genes involved in fairy ring formation have undetected low-level homology among species. Comparative genome analyses with the closely related fairy ring-forming fungi *Lepista nuda* and *Lepista personata*, the genomes of which have not been sequenced substantially yet, might enable effective screening of candidate genes. Metabolite analysis across those fungi could be helpful for such a gene screening.

### Data access from F-RINGS web database

We built a web database called ‘F-RINGS’ (http://bioinf.mind.meiji.ac.jp/f-rings/) to deliver the genome information resources of *L. sordida* (Supplementary Fig. 4). Three search functions (‘Keyword Search’, ‘BLAST Search’, and ‘GO Tree View’), a download page, and web links are included in F-RINGS. ‘Keyword Search’ lets users find genes of interest by any keyword. When keywords are included in gene identifiers or functional annotations, the gene will be listed in result pages. ‘BLAST Search’ allows users to search for homologous nucleotide or amino acid sequences from the genome assembly or coding sequences. ‘GO Tree View’ is a graphical search tool for genes with Gene Ontology annotations^32^. It allows interactive gene searches by browsing unspecified GO terms. F-RINGS currently provides the genome browsing features of JBrowse^33^. The genome assembly sequences and gene structural annotations can be viewed. The ‘Download’ page has web links to download the genome sequencing data, the genome assembly sequence file in fasta format, the genome annotation file in GFF format (version 3), predicted gene sequences in fasta format, and functional gene annotations in tab-delimited text files. The ‘Link’ page includes web links with existing public domains about fairy-ring formation, and NCBI domains about gene involved in the production of fairy chemicals. These are useful for in-house applications such as comparative genomics or transcriptome analyses in *L. sordida* and other evolutionarily related mushroom species.

## Methods

### Sequencing data source

Three types of genomic sequencing data of *L. sordida*, PE sequencing and MP sequencing reads by an Illumina GAIIx system (Illumina, Inc., San Diego, CA, USA), and PE pyrosequencing reads on a Roche GS FLX Titanium system (Roche Diagnostics, Basel, Switzerland) were used (deposited in the DDBJ Sequence Read Archive under accession number DRA003879). Mycelia of a *Lepista sordida* strain (National Institute of Technology and Evaluation No. NBRC 112841, Fig. 1B) were analyzed in this sequencing project.

To quantify expression levels of candidate genes in fairy ring forming-fungus, the RNA sequencing data set for *Paxillus involutus* (Genbank Sequence Read Archive, SRP132705) were downloaded and mapped onto all the predicted transcriptome sequences in *P. involutus* by BWA mem^34^ (ver. 0.7.15). Raw mapping data were converted into bam files using SAMtools^35^ (ver. 1.9), and the number of reads on each transcript sequence was counted and normalized into the reads per kilo base per million (RPKM) scale.

### Preprocessing of sequencing data

Redundant Illumina short reads, which showed perfect sequence identity among reads, were eliminated by an in-house Perl script. The adapter sequences were trimmed by the Cutadapt-1.5 tool^36^. The resultant reads were quality filtered with an in-house Perl script as described by Yamamoto *et al*.^37^. Briefly, low-quality bases at the sequence read ends with a quality value less than 10 were trimmed. Reads were removed that met any of the following criteria: read length shorter than 20 bp, average quality score per read less than 17, low-quality bases (quality value less than 10) per read more than 10% or reads containing one or more ambiguous nucleotide ‘N’s. Raw sequence data from the GS FLX were processed using sff_extract software 0.3.0 (http://bioinf.comav.upv.es/sff_extract/index.html) to trim the adapter sequences.

### Genome sequence assembly

Preprocessed sequence reads were used for hybrid assembly by the following multi-step procedure. The Illumina PE and GS FLX Titanium sequence reads were both assembled into contigs by the Newbler package (ver. 2.9, Roche Diagnostics) with the options‘-large’, ‘-het’ and ‘-scaffold’, and then the contigs were connected with Illumina MP short reads to generate genomic scaffolds by the SSPACE-STANDARD-3.0 program^38^. Finally, Illumina PE reads were used to fill an indefinite nucleotide ‘N’ in the genomic scaffolds by the GapCloser tool (ver. 1.12)^39^.

### *k*-mer analysis

A *k*-mer analysis was applied to the preprocessed Illumina short reads using the JELLYFISH tool^40^ with *k*-mer length of 17 (ver. 2.2.3). The distribution of *k*-mer frequencies was used for estimation of the genome size by the method described by Li and Waterman^41^. The software GCE^42^ (ver. 1.0.0), which is a genome size estimator utilizing the method based on a Bayesian model, was also used for genome size prediction.

### Genome annotation

For structural annotation, repeat sequences were first masked using the RepeatModeler (ver. 1.0.8) and RepeatMasker (ver. 4.0.3) programs (http://www.repeatmasker.org/RepeatModeler.html; http://www.repeatmasker.org). Then, protein coding genes were predicted by Augustus^43^ (ver. 3.0.3) with the option ‘–species = laccaria_bicolor’, SNAP^44^ (ver. 2006-07-28), and GeneMark-ES^45^ (ver. 3.48) using the self-training method with the default conditions. Transfer RNA (tRNA) genes were predicted by a search on the tRNAscan-SE server^46^ (ver. 1.3.1). Ribosomal RNA (rRNA) genes were predicted by the tools RNAmmer version 1.2 server^47^ and Infernal^48^ ver. 1.1.1. The presence of orthologues conserved among eukaryotes was checked by BUSCO^49^ (ver. 3.0.2) for evaluation of the genome assembly and gene annotations. Functional annotation was obtained by searches for homologous sequences. Specifically, predicted protein sequences in *L. sordida* were searched against the non-redundant protein database ‘nr’^50^ and the universal protein knowledgebase ‘UniProtKB’^51^ by the BLASTP program^52^ with a threshold e-value of 1e^−5^ and 50% query coverage. A KEGG Orthology search was carried out using the KEGG Automatic Annotation Server (KAAS)^53^ by the assignment method of ‘bi-directional best hit’ against data of any species in the KEGG database^54^. We conducted a sequence search by InterProScan^55^ to predict protein domains and assign Gene Ontology (GO) terms^56^. Eukaryotic orthologous group (KOG) categories^57^ were assigned by the WebMGA server^58^. Genes of carbohydrate-active enzymes, which act on lignocellulose degradation, were searched by dbCAN^59^ with an e-value of 1e^−3^ and a threshold of 50% of subject coverage.

### Protein sequence search in fungi

Deduced protein sequences were searched against the fungal genomics resource database MycoCosm^60^ by the BLASTP program with an e-value of 1e^−5^.

### Construction of phylogenetic dendrogram

We used the 1.7 kb small subunit of 18S ribosomal RNA gene sequences from 48 basidiomycete fungi for phylogenetic analysis. A dendrogram was constructed by UPGMA clustering^61^ after alignment by the ClustalW software^62^.

### Database building

The database F-RINGS was constructed on a Linux server with the CentOS-5.11 operating system (64-bit) using the LAMP stacks Apache 2.2.31, MySQL 5.0.95, and PHP 5.3.3. JBrowse version 1.12.1^33^ was implemented for browsing the draft genome sequence.

## Supplementary information

Supplementary Figures

Supplementary Table1

Supplementary Table2

Supplementary Table3

Supplementary Table4
