# Supplementary material for: Genome sequence analysis of the fairy ring-forming fungus Lepista sordida and gene candidates for interaction with plants
Source: Sci Rep. 2019 Apr 10;9:5888. doi: 10.1038/s41598-019-42231-9 (PMC6458111; doi:10.1038/s41598-019-42231-9)
Supplement: Supplementary file 1 — Supplementary Figures [file 41598_2019_42231_MOESM1_ESM.pdf]

Genome sequence analysis of the fairy ring-forming fungus *Lepista sordida* and gene candidates for interaction with plants

Tomoyuki Takano[1], Naoki Yamamoto[1†], Tomohiro Suzuki[2], Hideo Dohra[3], Jae-Hoon Choi[3,4], Yurika Terashima[4], Koji Yokoyama[1], Hirokazu Kawagishi[3,4,5*], Kentaro Yano[1*]

[1]Bioinformatics Laboratory, School of Agriculture, Meiji University, 1-1-1 Higashi-Mita, Kawasaki 214-8571, Japan

[2]Center for Bioscience Research and Education, Utsunomiya University, 350 Mine-machi, Utsunomiya, Tochigi 321-8505, Japan

[3]Research Institute of Green Science and Technology, Shizuoka University, 836 Ohya, Suruga-ku, Shizuoka 422-8529, Japan

[4]Graduate School of Integrated Science and Technology, Shizuoka University, 836 Ohya, Suruga-ku, Shizuoka 422-8529, Japan

[5]Graduate School of Science and Technology, Shizuoka University, 836 Ohya, Suruga-ku, Shizuoka 422-8529, Japan

[†]Present address: Rice Research Institute, Sichuan Agricultural University, 211 Huiminglu, Wenjiang, Chengdu, China

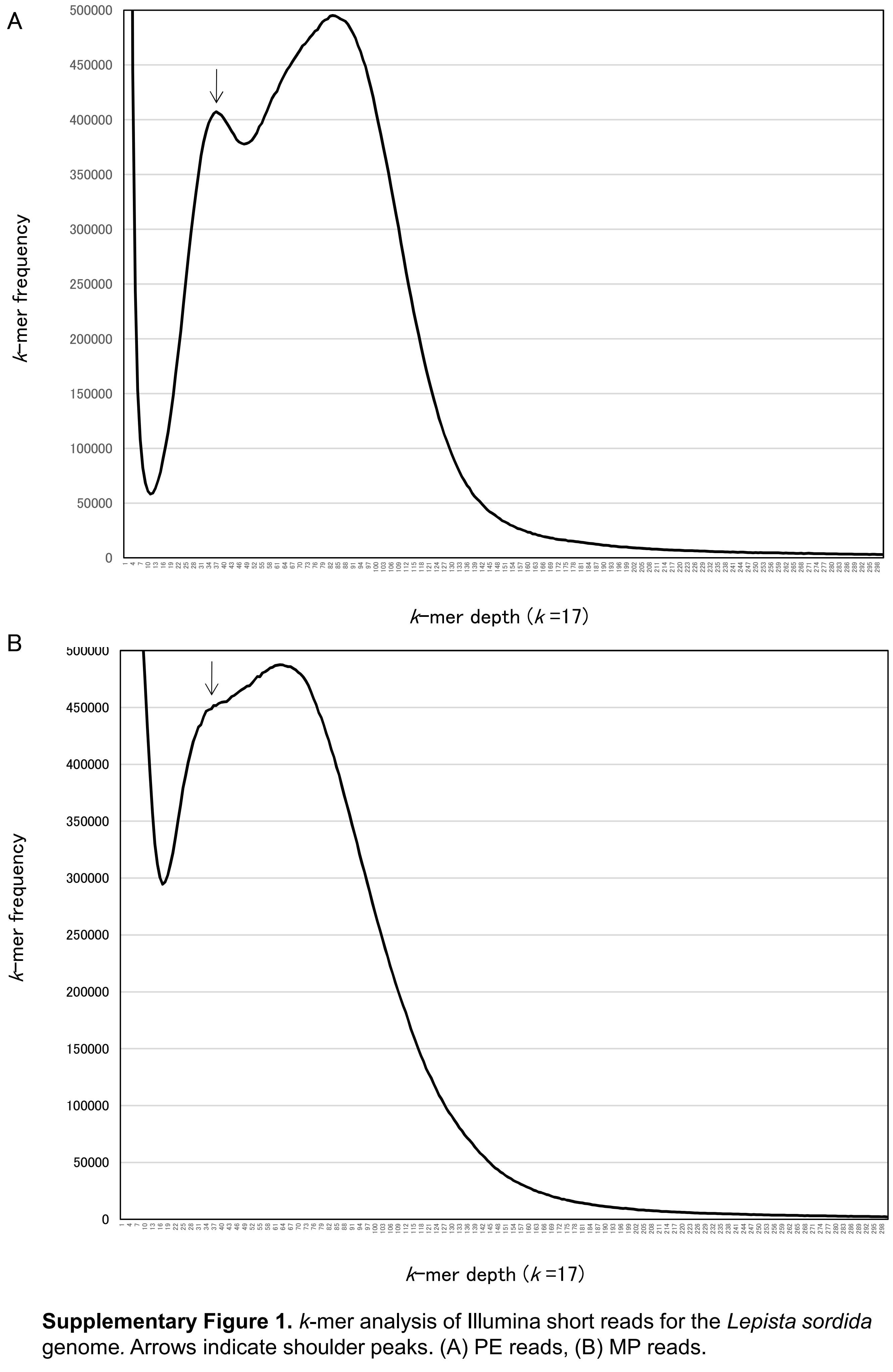

Supplementary Figure 1. *k*-mer analysis of Illumina short reads for the *Lepista sordida* genome. Arrows indicate shoulder peaks. (A) PE reads, (B) MP reads.

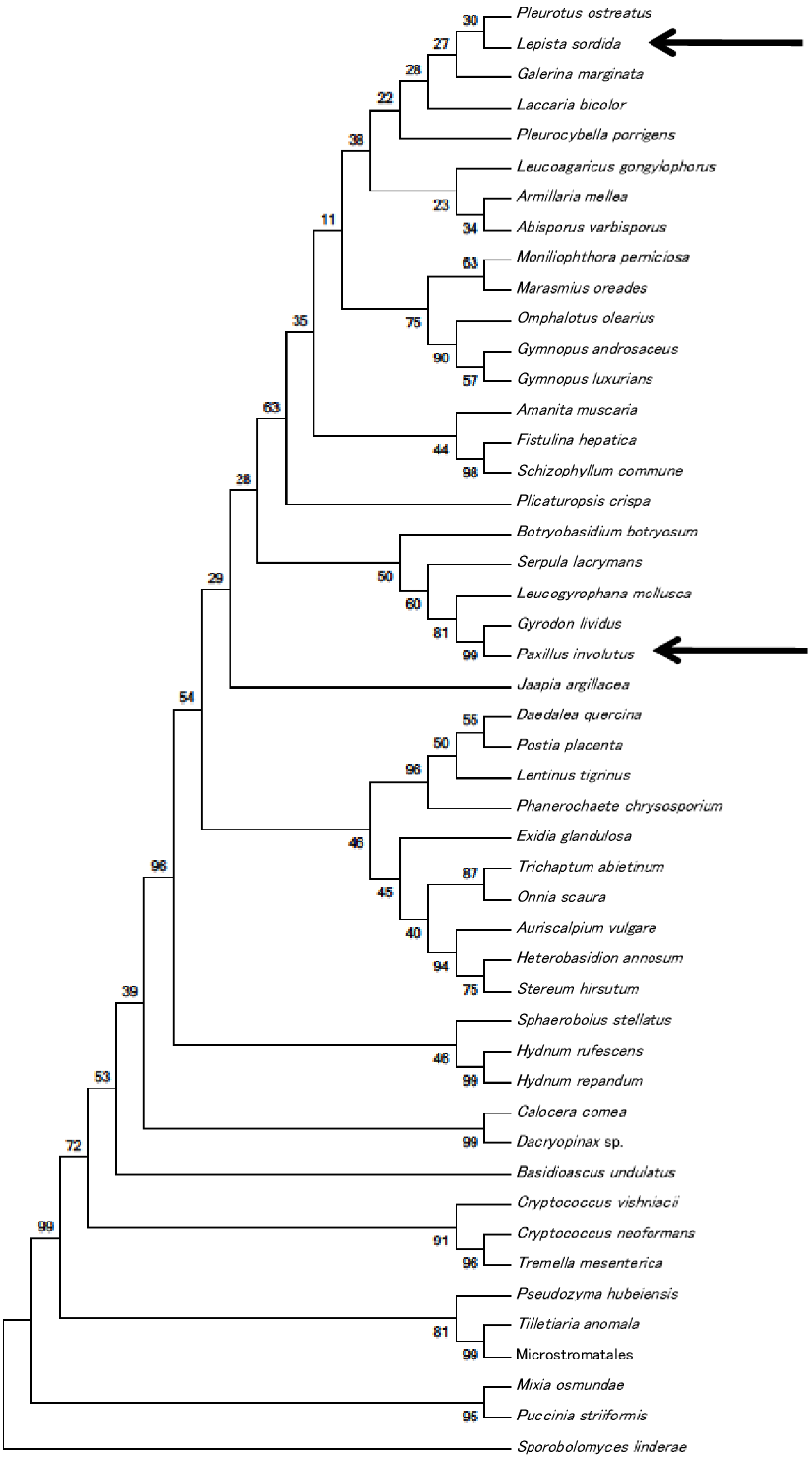

Supplementary Figure 2. Phylogenetic dendrogram of 48 basidiomycetes. The dendrogram was constructed by UPGMA clustering of the small subunit of 18S ribosomal RNA gene sequences. We used 1.7 kb sequences after alignment by the ClustalW software. Numerals indicate the bootstrap values in 100 trials.

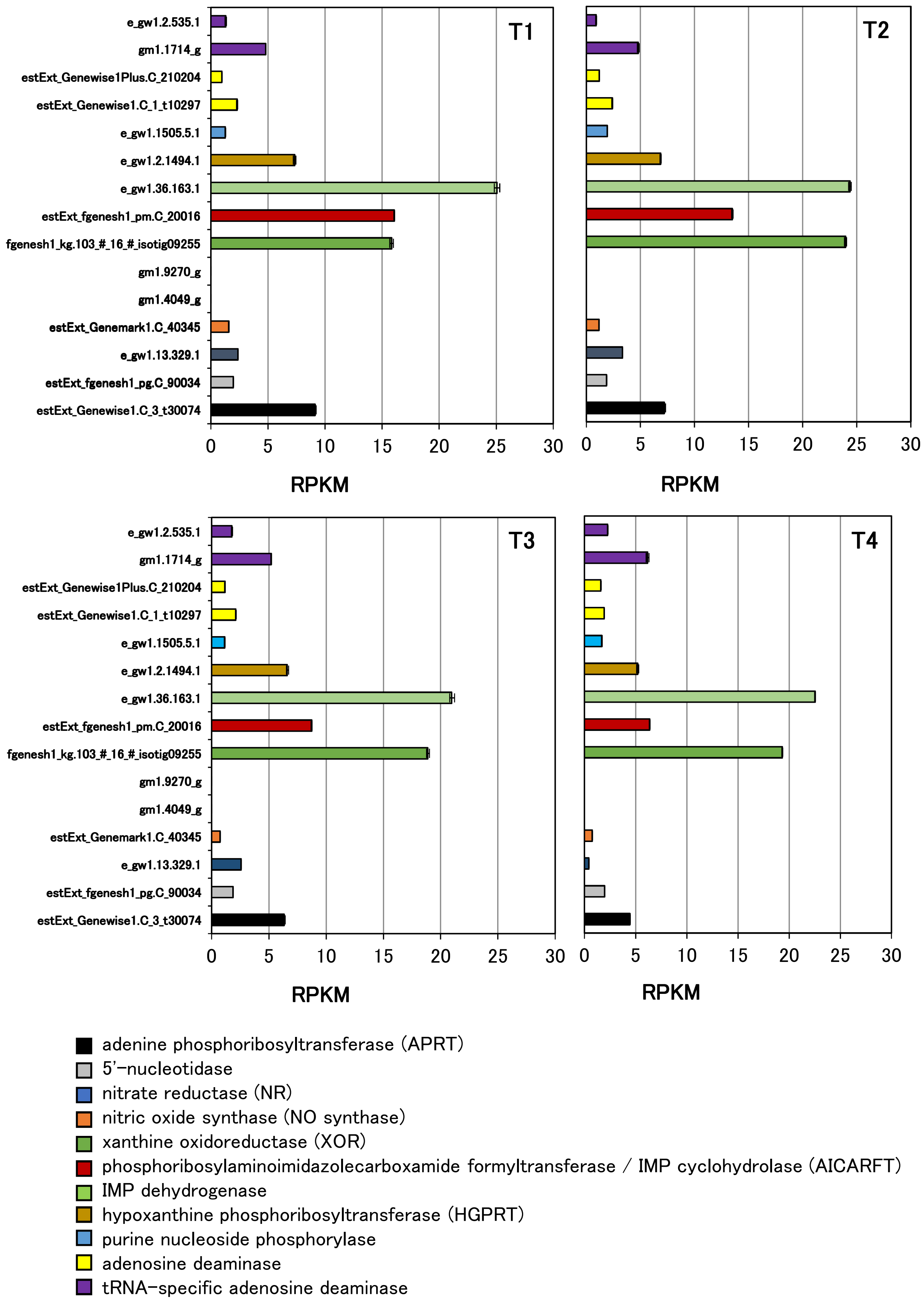

Supplementary Figure 3. Expression levels (RPKM) of candidates enzyme genes for biosynthesis of fairy chemicals in *P. involutus*. Error bars represent standard errors of three replicates in each time point (T1-T4).

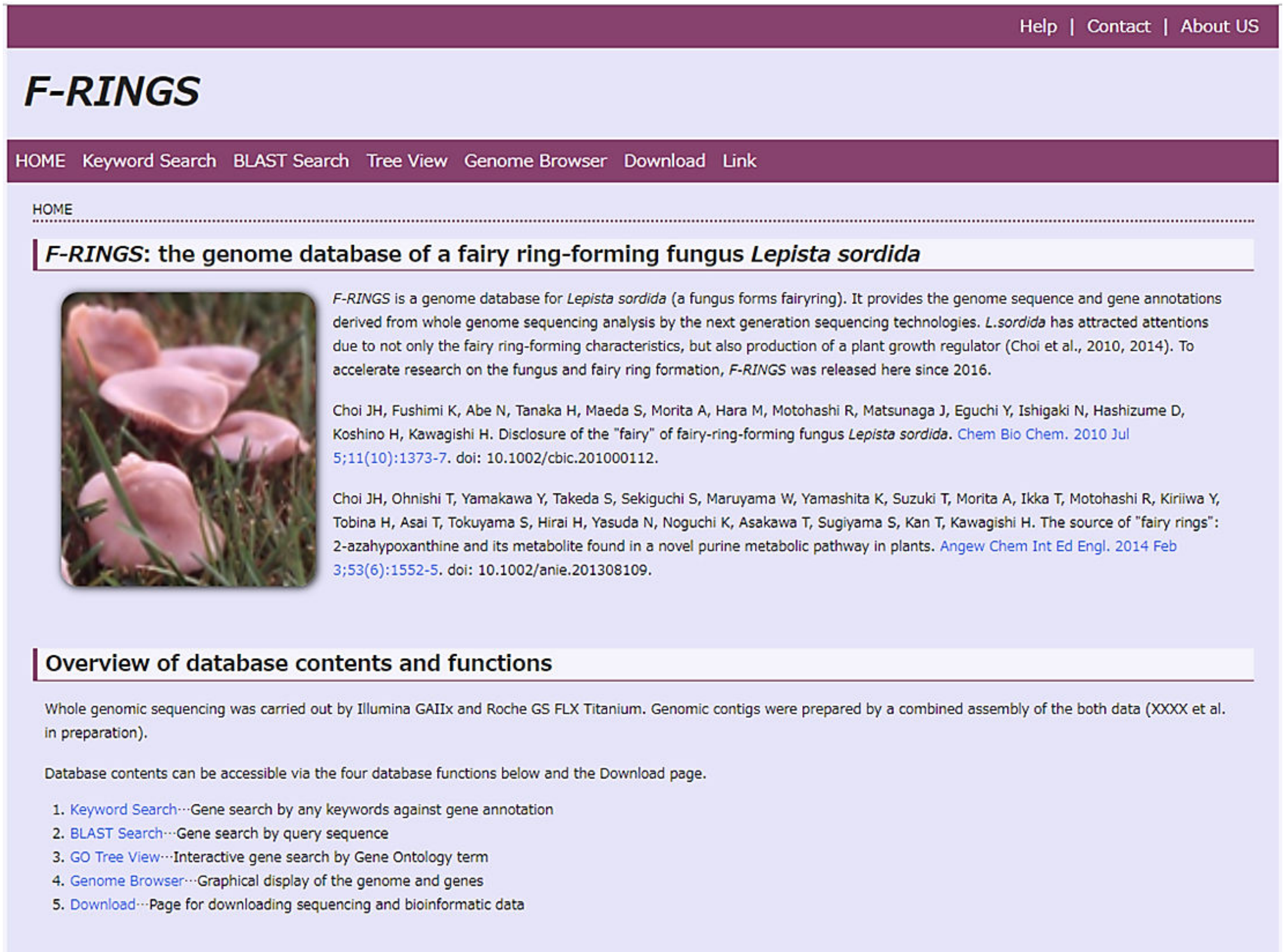

Help | Contact | About US

F-RINGS

HOME Keyword Search BLAST Search Tree View Genome Browser Download Link

HOME

F-RINGS: the genome database of a fairy ring-forming fungus *Lepista sordida*

F-RINGS is a genome database for *Lepista sordida* (a fungus forms fairyring). It provides the genome sequence and gene annotations derived from whole genome sequencing analysis by the next generation sequencing technologies. *L.sordida* has attracted attentions due to not only the fairy ring-forming characteristics, but also production of a plant growth regulator (Choi et al., 2010, 2014). To accelerate research on the fungus and fairy ring formation, *F-RINGS* was released here since 2016.

Choi JH, Fushimi K, Abe N, Tanaka H, Maeda S, Morita A, Hara M, Motohashi R, Matsunaga J, Eguchi Y, Ishigaki N, Hashizume D, Koshino H, Kawagishi H. Disclosure of the "fairy" of fairy-ring-forming fungus *Lepista sordida*. Chem Bio Chem. 2010 Jul 5;11(10):1373-7. doi: 10.1002/cbic.201000112.

Choi JH, Ohnishi T, Yamakawa Y, Takeda S, Sekiguchi S, Maruyama W, Yamashita K, Suzuki T, Morita A, Ikka T, Motohashi R, Kiriiwa Y, Tobina H, Asai T, Tokuyama S, Hirai H, Yasuda N, Noguchi K, Asakawa T, Sugiyama S, Kan T, Kawagishi H. The source of "fairy rings": 2-azahypoxanthine and its metabolite found in a novel purine metabolic pathway in plants. Angew Chem Int Ed Engl. 2014 Feb 3;53(6):1552-5. doi: 10.1002/anie.201308109.

Overview of database contents and functions

Whole genomic sequencing was carried out by Illumina GAIIx and Roche GS FLX Titanium. Genomic contigs were prepared by a combined assembly of the both data (XXXX et al. in preparation).

Database contents can be accessible via the four database functions below and the Download page.

1. Keyword Search···Gene search by any keywords against gene annotation
2. BLAST Search···Gene search by query sequence
3. GO Tree View···Interactive gene search by Gene Ontology term
4. Genome Browser···Graphical display of the genome and genes
5. Download···Page for downloading sequencing and bioinformatic data

Supplementary Figure 4. The top page of the genome database *F-RINGS*.
